# The Sight-Testing Question: A Suggested Compromise

**Published:** 1905-05-20

**Authors:** 


### The Hospital.

A Journal of

The Medical Sciences and Hospital Administration.

Vol. XXXVIII.—No. 973. SATURDAY, MAY 20, 1905.

### The Sight-testing Question: A Suggested Compromise.

Some of the professional journals devoted to the interests of ophthalmic science and practice are at present concerned with a proposal to approve an arrangement under which the estimation of errors of refraction and the prescription of glasses by retailers of spectacles may receive a quasi-official sanction. The suggestion is that advice to use glasses of any order shall, in the case of such persons, be restricted to patients in whom a full measure of vision either already exists or can be secured by spectacle lenses. In all other instances persons with defective sight are, it is to be presumed, to be referred to "those legally authorised to treat pathologic conditions and to take the full responsibility for the same." In this way, we are told, there may be found the "golden bridge desired alike by the ophthalmic surgeon and the better class of *boná-fide* opticians." This suggestion, be it noted, is commended by one of our contemporaries as "a moderate and business-like proposal." With this conclusion we are unable to agree. In the first place, the fact that a patient has a full measure of visual acuity is no guarantee of the absence of such serious forms of disease as optic neuritis, albuminuric retinitis, and commencing optic atrophy, and, in addition, the proposed test would permit many children with ciliary spasm to be condemned to wear entirely unsuitable glasses. Further, we object to any proposal as a result of which the medical profession may be made to share the responsibility for treatment conducted by unqualified persons. It may be true that a large number of individuals who complain of defective vision—presbyopes for example—need no further advice than can be readily obtained from the rule-of-thumb methods adopted by the seller of spectacles. But no experienced practitioner will deny that in accepting advice resting on such a basis there is a certain amount of risk. It is for the complaining member of the public and the spectacle vendor to shoulder that risk and to share it wholly between themselves.

If, in a more or less authoritative fashion, it be once allowed by the medical profession that the methods of the unqualified practitioner are safe provided their adoption gives a full standard of vision, the profession must inevitably be held responsible, or at least partially responsible, for the occasional catastrophes which will inevitably result. Again, if the principle advocated in this so-called "compromise" is to be recognised in connection with visual defects there can be no logical obstacle to its extension in other directions. The prescribing chemist and the bone-setter can, equally with the spectacle maker, claim a partial recognition on the ground that, according to the verdict of individual sufferers, their respective methods have proved competent to afford relief. All such proposals, in our judgment, tend to compromise the profession, and, what is more important, are antagonistic to the public interest. The teachings of experience indicate quite plainly that risk is only to be avoided by those who complain of impaired health by accepting guidance from advisers who are familiar with the facts of pathology and have practical familiarity with the beginnings of disease. That risks are often accepted with impunity is quite true. But that such conduct is either wise or safe is another and an entirely different proposition. If members of the public like to accept the risk of adopting advice in regard to questions of health from unqualified persons, that, it seems to us, is their own affair. But the medical profession, knowing what such risks may entail, ought in the public interest carefully to abstain from lending any sanction to such action. We would institute no measures to drive or compel the public to seek advice and help only in the consulting-rooms of the medical profession. When the law has taken steps to enable a clear and ready distinction to be drawn between properly qualified and unqualified medical advisers, the choice, so it seems to us, ought to rest with the public. It ought not to be the ambition of the profession to apply compulsion to any member of the public. As good citizens we may well desire that the public shall not be deceived in reference to medical advice, and shall not be made the dupes of mock diplomas and misleading titles. But this once secured there is no need for further concern. To attempt to limit individual choice and to suppress by legal penalties what is commonly called unqualified practice, seem to us ends neither desirable nor practicable. So long as the spectacle makers do not assume titles which mislead the public, the relationship between themselves and their customers is their own affair. If they do assume such titles, medical practitioners like other citizens are entitled to protest. Short of this, they and their clients must arrange their own affairs. Certainly in such transactions the medical profession if true to its best traditions and to the public interest can have neither responsibility nor share.

